# Do general practitioners prescribe more antimicrobials when the weekend comes?

**DOI:** 10.1186/s40064-015-1505-6

**Published:** 2015-11-24

**Authors:** Meera Tandan, Sinead Duane, Akke Vellinga

**Affiliations:** Discipline of General Practice, School of Medicine, National University of Ireland Galway, 1 Distillery Road, Galway, Ireland; Discipline of Bacteriology, School of Medicine, National University of Ireland Galway, Galway, Ireland

**Keywords:** Antimicrobial prescribing, General practice, Weekend

## Abstract

Inappropriate antimicrobial prescribing contributes to the global spread of antimicrobial resistance. The pending weekend with changed availability of general practitioners (GP) and increased patient concern may increase the intention to prescribe antimicrobials. The aim of this study is to analyse variation in antimicrobial prescribing between weekdays and weekend in Irish general practice. All prescribing data over a 15 month period was obtained from the 30 practices participating in the Supporting the Improvement and Management of Prescribing for urinary tract infection (SIMPle) study. Antimicrobials were classified using anatomical therapeutic chemical classification code guidelines. Prescribing of antimicrobials per total number of prescriptions was compared between weekdays (Monday to Thursday) and the weekend (Friday to Sunday). Antimicrobials were generally more often prescribed during weekends; the antimicrobial prescribing rate was greater by 9.2 % on Friday compared to average prescribing on other weekdays (21.4 vs. 19.6 %). The chance of an antimicrobial prescription was 1.07 (95 % CI 1.04–1.10) higher on weekend days compared to weekdays. This was reflected in increased prescriptions for ampicillin, co-amoxiclav, nitrofurantoin, quinolones and macrolides. However, if antimicrobials were prescribed, no significant differences were observed between weekdays and weekend among the different classes of antimicrobials. GPs prescribe relatively more antimicrobials during the weekend compared to weekdays. However, the patterns of antimicrobial prescribing did not differ according to the day of prescription.

*Trial Registration* The intervention was registered with ClinicalTrials.gov on 26 July 2013, ID number NCT01913860

## Background

Antimicrobial resistance (AMR) is a global health threat (WHO 2014a). Inappropriate consumption of antimicrobials is well known as a cause of the spread of resistance (Bronzwaer et al. 2002). Approximately 80 % of the antimicrobials are prescribed in general practice, mainly for urinary tract infections (UTI) and bronchial infections and conditions (Murphy et al. 2011; Hulscher et al. 2010). A study conducted in Ireland suggests that antimicrobials are overprescribed in general practice and 80 % of the general practitioners (GPs) acknowledged overprescribing, mainly due to the (perceived) demand from patients (Cotter and Daly 2007).

GPs are the first medical point of contact for individuals feeling ill and they play an important role in health. Studies have shown variation in the prescribing of antimicrobials with higher prescribing on Fridays compared to other weekdays (Kuehlein et al. 2010; Huibers et al. 2014). On Saturday and Sunday, primary care services operate an out-of-hours service. However, in recent years, the provision of out-of-hours service has been a debatable issue because of additional stress to GPs. In Ireland, each county has organised their own service by establishing cooperatives, which enable GPs to spend less time on call by working within a large rota (Lynch 2004).

Understanding of prescribing patterns may open opportunities for interventions to improve the quality of prescribing. The aim of this study was to understand prescribing patterns of antimicrobials on various days of the week and to assess whether there was significance difference in antimicrobial prescribing on weekday and weekend in Irish general practices.

## Methods

### Setting

In 2013, 30 general practices participated in the Supporting the Improvement and Management of Prescribing for urinary tract infection (SIMPle) study (Duane et al. 2013). This cluster intervention in general practice aimed to address the quality of prescribing for UTI. Remote, electronic data collection was initiated through the Irish Primary Care Research Network (IPCRN), a national research network of general practices established to enhance research and provide new tools to audit and manage patient care. Control and intervention arms received a workshop on consultation coding. The intervention arms received a workshop on appropriate prescribing for UTI supported by a practice specific audit report. Anonymised data on all consultations over a 15 month period were uploaded by the participating practices.

### Patients

A total of 129,634 acute prescriptions from 30 general practices were recorded from June 2013 to August 2014 (15 months). 33,097 of these were antimicrobial prescriptions for adult patients. The IPCRN provided data on patients’ age, gender, medical card status, consultation date, type of prescription, and Anatomical Cherapeutic Chemical (ATC) coding. All prescriptions were categorised based on the WHO-ATC level-5 codes (WHO 2014b). A medical card provides the holder with free healthcare and medication. Entitlement to a medical card is based on income and age and about one-third of the population under 70 have a medical card and 97 % of those aged 70 and older (Health Insurance Authority 2014).

### Statistical analysis

Descriptive analyses of basic characteristics such as age, gender, medical card status and antimicrobial prescription were presented in percentages as well as mean and median of age. The prescription proportions of antimicrobials on different days were calculated by dividing the number of antimicrobial prescribed by total number of prescriptions on each weekday (only one prescription per consultation was included). If an antimicrobial was prescribed this was prioritised and when more than one antimicrobial was prescribed, narrow spectrum antimicrobials were prioritised. Prescriptions made on Friday/Saturday/Sunday were categorised as weekend and compared with the prescriptions made on Monday to Thursday (Huibers et al. 2014). Both the relative antimicrobial prescriptions (per total number of prescriptions) and the antimicrobial class (per total number of antimicrobial prescriptions) were compared between the weekend and weekdays (Monday to Thursday). A mixed model approach correcting for clustering at the practice level (xtgee) was applied including confounding factors (age, gender, medical card and intervention arm). The results were presented as odds ratios (OR) and associated 95 % confidence interval (CI). Overall statistical analysis was performed with IBM SPSS v21.0 and STATA version 13.0.

## Results

### Overview of the prescriptions

A total of 33,097 patients (25.5 %, from 129,634 prescriptions) received an antimicrobial prescription during consultation. Females accounted for 62.7 % of all prescriptions and 69.5 % of all prescriptions were to medical card holders (Table 1). Similarly, the percentage antimicrobials prescribed was higher for females (65.4 %) compared to males and for medical card patients (61.6 %). The mean age of the patients who received a prescription was 52.3 (±20.1) years and age differed significantly between those who did get an antimicrobial (51.7, ±20.8) and those who received any other prescription (52.5, ±19.9) years. Of all the antimicrobial prescriptions, ampicillin (AMP) was most often prescribed (38.5 %) followed by co-amoxiclav (AMC, 19.8 %), macrolides (12.9 %) and nitrofurantoin (12.3 %).

**Table 1:** Overview of prescriptions

Variables	All prescriptions (N = 129,624) %	Antimicrobial prescriptions (N = 33,097) %
**Gender**		
Female	62.7	65.3
Male	37.1	34.6
Unknown	0.2	0.2
**Age (years)**	Mean = 52.3, Med = 52, SD = ±20.1	Mean = 51.7, Med = 51, SD = ±20.8
17–25	10.5	12.5
26–50	37.9	37.1
51–75	36.0	34.2
>75	15.6	16.3
**Arms**		
Intervention arm-1	35.4	37.1
Intervention arm-2	36.5	33.2
Control arm	28.0	29.7
**Medical card type**		
GMS*	69.5	61.6
Private	30.5	38.4
**Antimicrobials prescribing**		
AMP	9.8	38.5
AMC	5.1	19.8
Macrolides	3.3	12.9
Nitrofurantoin	3.1	12.3
Quinolone	1.1	4.5
Tetracycline	1.0	3.8
Cephalosporin	0.8	3.3
Trimethoprim	0.7	2.8
Other	3.7	14.6

* *GMS* medical card holder patients

### Antimicrobial prescriptions on weekdays and weekend

**Fig. 1:**
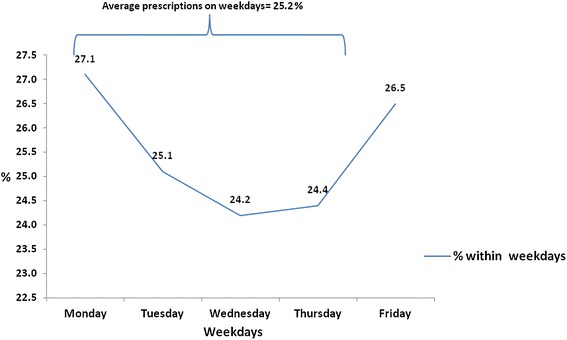
Antimicrobials prescribing based on the total prescription made on the different weekdays

There was variation in the proportion of antimicrobial prescription on different days of the weekdays (Fig. 1). Of all antimicrobials prescribed, 22.5 % were prescribed on Monday, 20.7 % on Tuesday, 17.1 % on Wednesday, 18.1 % on Thursday and 21.4 % from Friday on (not shown in figure). Relative to all prescriptions, the percentage of antimicrobials was 25.2 % on weekdays compared to 26.5 during the weekend. The percentage was higher on Mondays (27.1 %) and Fridays (26.5 %) compared with the other days.

After correcting for other factors, the chance of an antimicrobial prescription was significantly higher during the weekend days compared to weekdays (OR = 1.07, 95 % CI 1.04–1.10) (Table 2). The higher prescribing during the weekend was mainly due to higher prescription of ampicillin (OR = 1.06, 95 % CI 1.01–1.11) and nitrofurantoin (OR = 1.18, 95 % CI 1.09–1.27) of all prescriptions (not shown in table). Of all prescriptions, significant differences were not observed in prescribing co-amoxiclav, trimethoprim, quinolone, tetracycline, cephalosporin and macrolides between weekdays and weekend. When an antimicrobial was prescribed, this was significantly more likely to be nitrofurantoin (OR = 1.12, 95 % CI 1.03–1.21) or ampicillin (OR = 1.06, 95 % CI 1.01–1.11) during the weekend while less likely to be co-amoxiclav (OR = 0.89, 95 % CI 0.84–0.96). There was no significant difference in other class of antimicrobials prescribing in weekdays and weekend in terms of antimicrobials prescribed (Table 2).

**Table 2:** GEE model for antimicrobial prescribing

Variables	AB** prescribing			Nitrofurantoin			AMP		
	OR	95 % CI		OR	95 % CI		OR	95 % CI	
**Weekdays vs. weekend**									
Weekdays	Reference			Reference			Reference		
Weekends	1.07	1.04	1.10	1.12	1.03	1.21	1.06	1.01	1.11
**Gender**									
Female	Reference			Reference			Reference		
Male	0.86	0.83	0.88	0.20	0.18	0.22	1.12	1.08	1.16
** Age**	1.00	0.99	1.00	1.01	1.01	1.02	0.99	0.99	0.99
**Medical card type**									
Private	Reference			Reference			Reference		
GMS*	0.61	0.59	0.63	1.15	1.07	1.24	0.72	0.69	0.75
**Arms**									
Control arm	Reference			Reference			Reference		
Intervention arm-1	0.79	0.77	0.82	1.05	0.97	1.13	0.77	0.74	0.80
Intervention arm-2	0.98	0.95	1.01	0.93	0.86	1.01	0.89	0.86	0.94

Variables	AMC			Trimethoprim			Quinolone		
	OR	95 % CI		OR	95 % CI		OR	95 % CI	
**Weekdays vs. weekend**									
Weekdays	Reference			Reference			Reference		
Weekends	0.89	0.84	0.96	1.04	0.89	1.22	1.06	0.94	1.21
**Gender**									
Female	Reference			Reference			Reference		
Male	1.20	1.13	1.27	0.37	0.31	0.44	1.82	1.64	2.02
** Age**	1.00	0.99	1.00	1.01	1.01	1.01	1.02	1.02	1.02
**Medical card type**									
Private	Reference			Reference			Reference		
GMS*	0.82	0.76	0.86	1.40	1.20	1.63	1.14	1.01	1.27
**Arms**									
Control arm	Reference			Reference			Reference		
Intervention arm-1	0.93	0.87	0.99	1.13	0.96	1.33	0.97	0.86	1.12
Intervention arm-2	0.91	0.86	0.98	1.13	0.96	1.33	1.02	0.91	1.16

Variables	Cephalosporin			Tetracycline			Macrolide		
	OR	95 % CI		OR	95 % CI		OR	95 % CI	
**Weekdays vs. weekend**									
Weekdays	Reference			Reference			Reference		
Weekends	0.95	0.81	1.10	1.08	0.95	1.24	0.95	0.88	1.03
**Gender**									
Female	Reference			Reference			Reference		
Male	0.83	0.73	0.96	1.05	0.93	1.18	1.16	1.09	1.24
** Age**	1.01	1.01	1.01	0.99	0.99	0.99	1.00	1.00	1.00
**Medical card**									
Private	Reference			Reference			Reference		
GMS*	1.04	0.91	1.18	0.73	0.65	0.82	0.82	0.77	0.88
**Arms**									
Control arm	Reference			Reference			Reference		
Intervention arm-1	1.41	1.22	1.65	1.06	0.92	1.22	1.20	1.11	1.30
Intervention arm-2	1.50	1.29	1.76	1.27	1.11	1.46	1.24	1.14	1.34

** *AB* antimicrobials, * *GMS* medical card holder patients

In general, males were less likely to get an antimicrobial prescription (OR = 0.86, 95 % CI 0.83–0.88), and older age did not increase the odds of an antimicrobial prescription. Medical card patients were significantly less likely to receive an antimicrobial prescription during a consultation 0.61 (0.59–0.63). When patients received a prescription for an antimicrobial, differences were observed in the chance of receiving a prescription for each antimicrobial separately. Generally, nitrofurantoin and trimethoprim were more often prescribed for medical card and older patients, but less often in males, while ampicillin and co-amoxiclav showed the opposite, less often prescribed in medical card and older patients and more often in males. Quinolones were more often prescribed for medical card patients, older patients and males. For tetracycline and macrolides, both were less often prescribed to patients with medical cards (Table 2).

When looking at the days separately, there was no difference between the weekend and Monday, but antimicrobial prescribing was significantly less on Tuesday, Wednesday and Thursday. This difference was due to the relative higher number of prescriptions for co-amoxiclav (OR = 1.18, 95 % CI 1.08–1.26) on Monday (not shown in table).

## Discussion

### Summary of main findings

Antimicrobials were more often prescribed during the weekend compared to weekdays. There was no difference in the antimicrobial prescribing on the different days of a week. Higher prescribing on Mondays may be due to the higher number of consultations of patients who could not access their own GP during the weekend and decided to wait till normal office hours. The increase in prescribing of antimicrobials in the weekend was largely due to higher prescribing of ampicillin and nitrofurantoin but co-amoxiclav was prescribed less often on weekends. The prescribing of other antimicrobials (trimethoprim, quinolones, tetracycline, cephalosporin and macrolides) remains more or less the same on weekend and weekdays.

### Strengths and limitations

The data used in this study were obtained through a direct download from the practice’s patient management software which was facilitated through IPCRN and are therefore reliable and complete. However, we have no information as to whether these prescriptions were then dispensed and consumed. In particular, we cannot determine whether any prescriptions were ‘delayed prescriptions’ which has been shown to significantly reduce antimicrobial consumption (Little et al. 2014). The practices included in this study were involved in an intervention to improve prescribing. The potential bias of the intervention has been included as a variable in the analysis but may have impacted antimicrobial prescribing in general compared to practices not in the study. However, it is not expected that the intervention itself influenced the outcome observed for this analysis as a separate analysis of the control group did not show any different conclusions.

We only had access to prescribing data; information on the reason for the GP visit or consultation codes was incomplete. The data used in this study originated from 30 participating practices which had a limited geographical spread for practical reasons. We have however no reason to assume that these practices differ from practices in the rest of Ireland, apart from the laboratory they use for the analysis of urine samples.

### Comparison with existing literature

Few studies have tried to explore the proportion of antimicrobial prescribing on different days of a week however the difference in prescribing on weekdays and weekends on different class of antimicrobial are yet remained to explore. Kuehlein et al. showed that prescribing was higher on Fridays (23 %) and nearly 2.5 times higher than the findings of our study (9.1 % higher on Friday than any other weekdays). The results were not explicit about differences in prescribing various types of antimicrobials between weekdays and weekends, but explored the difference in patients’ visits as per diagnosis (Kuehlein et al. 2010). In Denmark, the antimicrobial prescription proportion was also higher for weekends than on weekdays however no significant differences were observed or reported. The focus of this study was on the distribution of types of antimicrobials prescribed according to three different contact types; telephone consultation, clinical consultation and home visits (Huibers et al. 2014). A cohort study in Irish general practice showed similar findings to our study, with a higher proportion of prescribing during the weekend and additionally that private patients were more likely to receive antimicrobials than medical card patients (Murphy et al. 2011). Other possible explanatory factors of differences in antimicrobial prescribing between weekdays and weekends, as outlined in another paper (Blommaert et al. 2014), included differences in contact time or socio-economic factors, could not be further explored in our study due to data limitations.

### Implications for research and/or practice

The findings of the study suggest that the antimicrobial prescribing was higher on weekend as compared to other days of the week. Nitrofurantoin and ampicillin were most frequently prescribed. Knowing the prescribing of different types of antimicrobials on weekend compared to weekdays has no direct impact on antimicrobial resistance. However, it helps to understand prescribing patterns of antimicrobials in general practice, which could provide important information on overprescribing which is one of the causes of antimicrobial resistance and assist in designing complex interventions in the future.

## Conclusion

No differences were observed in the proportion of antimicrobials prescribed between the different days of the week. However, relative to all prescriptions, antimicrobial prescribing was higher on Mondays and Fridays compared to the other working days. This relative higher percentage just before and after the weekend is most likely due to limited access to GPs during the weekend.

## Abbreviations

AMR: Antimicrobial resistance
AMP: Ampicillin
AMC: Co-amoxiclav
ATC: Anatomical therapeutic chemical
CI: Confidence interval
GPs: General practitioners
IPCRN: Irish Primary Care Research Network
OR: Odds ratio
SIMPle: Supporting the Improvement and Management of Prescribing for urinary tract infection
SPSS: Statistical package for social science
UTI: Urinary tract infection
WHO: World Health Organisation
